# Supplementary material for: Identification of Freezing-Responsive microRNAs and Their Targets in Chinese Jujube by Small RNA and Degradome Sequencing
Source: Int J Mol Sci. 2026 Sep 4;27(17):7912. doi: 10.3390/ijms27177912 (PMC13566890; doi:10.3390/ijms27177912)
Supplement: Supplementary file 1 [file ijms-27-07912-s001.zip › Supplementary Material S2/novel_miR_68.pdf]

Provisional ID	:	novel_miR_68
Score total	:	1
Score for star read(s)	:	-1.3
Score for read counts	:	0
Score for mfe	:	0.7
Score for randfold	:	1.6
Score for cons. seed	:	
Total read count	:	789
Mature read count	:	643
Loop read count	:	146
Star read count	:	0

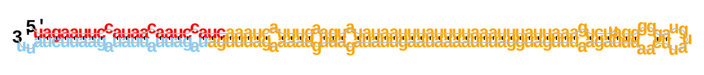

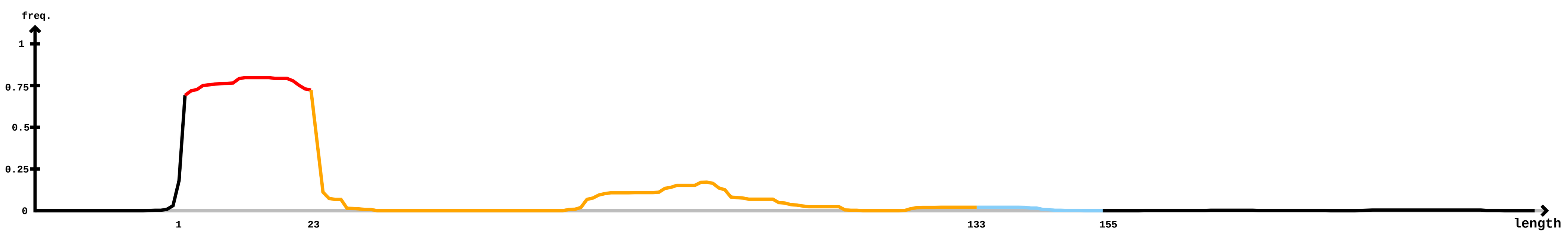


```
                                          Mature                                                                                                              Star
5'- uuauaaauuccuaauggaguuuauagaauuccauaacaauccaucaaaucauuugaaguauauaauuuauuuuaaauuuauuaaagucuuaggggauguauucaauuuagaauuugauggauuuaaaauaaguuauagauuguaaaagauuugauagauuauuauagaauucuauugauuccauaaagauuuuauaagaauccaacgaaaucuuucguuuuaaggcugaauuuuauauuaacaauuuuc  -3'   exp
    ((((((.((...(((((((.((((((((.((((.((((.(((((((.((((.(((.((((((((((((((((((((((((.(((.(((..((.....))..)))))).))))))))))))))))))))))))).))).)))).)))))))).)))).)))).))))))))).))))))))...)).)))))))....((((((((....))))))....)).........................   reads   mm   sample
    .................aguuuGuagaauuccauaaca...................................................................................................................................................................................................................   1   1   LT-1
    ..................guuuGuagaauuccauaacaauc................................................................................................................................................................................................................   1   1   LT-1
    ....................uuGuagaauuccauaaca...................................................................................................................................................................................................................   3   1   LT-2
    ....................uuGuagaauuccauaacaauc................................................................................................................................................................................................................   1   1   LT-1
    ....................uuauagaauuccauaacaaCcc...............................................................................................................................................................................................................   1   1   LT-1
    .....................uauagaauuccauaacaaucc...............................................................................................................................................................................................................   1   0   LT-1
    .....................uauagaauuccauaacaaucca..............................................................................................................................................................................................................   1   0   LT-3
    .....................uauagaauuccauaacaauccauc............................................................................................................................................................................................................   1   0   LT-1
    .....................uauagaauuccauaacaauccauc............................................................................................................................................................................................................   1   0   JCK-2
    .....................uauagaauuUcauaacaauccauc............................................................................................................................................................................................................   6   1   JCK-3
    .....................uauagaauuUcauaacaauccauc............................................................................................................................................................................................................   4   1   JCK-2
    .....................uauagaauuccauaacaauccauc............................................................................................................................................................................................................   1   0   JCK-1
    .....................uauagaauuUcauaacaauccauc............................................................................................................................................................................................................   2   1   JCK-1
    .....................uauagaauuccauaacaauccauca...........................................................................................................................................................................................................   1   0   LT-2
    ......................auagaauuUcauaacaaucc...............................................................................................................................................................................................................   2   1   LT-2
    ......................auagaauuccauaacaaucc...............................................................................................................................................................................................................   1   0   LT-2
    ......................auagaauuUcauaacaaucca..............................................................................................................................................................................................................   2   1   JCK-3
    ......................auagaauuUcauaacaaucca..............................................................................................................................................................................................................   3   1   JCK-1
    ......................auagaauuUcauaacaaucca..............................................................................................................................................................................................................   1   1   JCK-2
    ......................auagaauuccauaacaaucca..............................................................................................................................................................................................................   3   0   JT-1
    ......................auagaauuccauaacaauccaC.............................................................................................................................................................................................................   4   1   LT-2
    ......................auagaauuccauaacaauccauc............................................................................................................................................................................................................   21  0   LCK-2
    ......................auagaauuccauaacaauccauc............................................................................................................................................................................................................   1   0   LT-1
    ......................auagaauuccauaacaauccauc............................................................................................................................................................................................................   7   0   JCK-2
    ......................auagaauuUcauaacaauccauc............................................................................................................................................................................................................   1   1   JCK-3
    ......................auagaauuUcauaacaauccauc............................................................................................................................................................................................................   1   1   LT-2
    ......................auagaauuccauaacaauccGuc............................................................................................................................................................................................................   1   1   LT-3
    ......................auagaauuccauaacaauccauc............................................................................................................................................................................................................   2   0   LT-3
    ......................auagaauuUcauaacaauccauc............................................................................................................................................................................................................   4   1   JCK-2
    ......................auagaauuccauaacaauccauc............................................................................................................................................................................................................   4   0   JCK-1
    ......................auagaauuccauaacaauccauc............................................................................................................................................................................................................   16  0   LCK-1
    ......................auagaauuccauaacaauccauc............................................................................................................................................................................................................   10  0   JCK-3
    ......................auagaauuUcauaacaauccauc............................................................................................................................................................................................................   1   1   LT-1
    ......................auagaauuccauaacaauccauca...........................................................................................................................................................................................................   5   0   JCK-1
```

```
                       Mature                                                                                                                                    Star
uuauaaauuccuaauggaguuuauagaauuccauaacaauccaucaaaucauuugaaguauauaauuuauuuuaaauuuauuaaagucuuaggggauguauucaauuuagaauuugauggauuuaaaauaaguuauagauuguaaaagauuugauagauuauuauagaauucuauugauuccauaaagauuuuauaagaauccaacgaaaucuuucguuuuaaggcugaauuuuauauuaacaauuuuc
......................auagaauuccauaacaauccauca...........................................................................................................................................................................................   10   0   JCK-2
......................auagaauuccauaacaauccaAca...........................................................................................................................................................................................   1    1   JCK-1
......................auagaauuccauaacaauccGuca...........................................................................................................................................................................................   1    1   LT-1
......................auagaauuUcauaacaauccauca...........................................................................................................................................................................................   2    1   JCK-3
......................auagaauuUcauaacaauccauca...........................................................................................................................................................................................   3    1   JCK-1
......................auagaauuccauaacaauccaAca...........................................................................................................................................................................................   1    1   JCK-2
......................auagaauuccauaacaauccaAca...........................................................................................................................................................................................   1    1   LT-1
......................auagaauuccauaacaauccauca...........................................................................................................................................................................................   5    0   LCK-3
......................auagaauuccauaacaauccauca...........................................................................................................................................................................................   1    0   LT-3
......................auagaauuccauaacaauccauca...........................................................................................................................................................................................   8    0   JCK-3
......................auagaauuccauaCcaauccauca...........................................................................................................................................................................................   1    1   LCK-3
.......................uagaauuccauaacaauc................................................................................................................................................................................................   8    0   LCK-3
.......................uagaauuccauaacaauc................................................................................................................................................................................................   2    0   LT-3
.......................uagaauuccauaacaaucc...............................................................................................................................................................................................   9    0   LT-2
.......................uagaauuccauaacaaucc...............................................................................................................................................................................................   5    0   LT-3
.......................uagaauuccauaacaaucU...............................................................................................................................................................................................   2    1   LT-2
.......................uagCauuccauaacaaucc...............................................................................................................................................................................................   1    1   LT-2
.......................uagaauuccauaacaauccC..............................................................................................................................................................................................   1    1   LT-1
.......................uagaauuccauaacaaucca..............................................................................................................................................................................................   2    0   LT-1
.......................uagaauuccauaacaaucca..............................................................................................................................................................................................   3    0   LT-3
.......................uagaauuccauaacaauccau.............................................................................................................................................................................................   1    0   JCK-3
.......................uagaauucUauaacaauccau.............................................................................................................................................................................................   1    1   LT-3
.......................uagaauuccauaacaauccauc............................................................................................................................................................................................   41   0   LCK-1
.......................uagaauuccauaacaauccauU............................................................................................................................................................................................   1    1   LT-3
.......................uagaauuccauaacaauccCuc............................................................................................................................................................................................   1    1   LCK-1
.......................uagaauuccauaacaGuccauc............................................................................................................................................................................................   1    1   LT-3
.......................uagaauuccauaacaauccauc............................................................................................................................................................................................   5    0   JCK-3
.......................uagaauucUauaacaauccauc............................................................................................................................................................................................   1    1   LT-3
.......................uagaauuccauaacaauccauc............................................................................................................................................................................................   15   0   LT-1
.......................uagaauuccauaacaauccauc............................................................................................................................................................................................   7    0   LT-2
.......................uagaauucUauaacaauccauc............................................................................................................................................................................................   2    1   LCK-3
.......................uagaauuccauaacaauccauc............................................................................................................................................................................................   6    0   LCK-3
.......................uagaauuccauaacaauccauU............................................................................................................................................................................................   1    1   JCK-3
.......................uagCauuccauaacaauccauc............................................................................................................................................................................................   1    1   JCK-2
.......................uCgaauuccauaacaauccauc............................................................................................................................................................................................   1    1   LT-3
.......................uagaauuccauaacaauccauc............................................................................................................................................................................................   49   0   LCK-2
.......................uagaauuccauaacUauccauc............................................................................................................................................................................................   2    1   LT-2
.......................uagaauuccauaacaauAcauc............................................................................................................................................................................................   2    1   JCK-2
.......................uagaauuccauaacaauAcauc............................................................................................................................................................................................   1    1   LT-3
.......................uagaauuccauaGcaauccauc............................................................................................................................................................................................   1    1   LT-3
.......................uagaauucUauaacaauccauc............................................................................................................................................................................................   2    1   JCK-3
.......................uagaauuccauaacaauAcauc............................................................................................................................................................................................   2    1   JCK-1
.......................uagaauuccauaacaauAcauc............................................................................................................................................................................................   1    1   JCK-3
.......................uagaauucUauaacaauccauc............................................................................................................................................................................................   3    1   JCK-2
.......................uagaauuccauaacaauccauc............................................................................................................................................................................................   6    0   JCK-1
.......................uagaauuccauaacaauccauc............................................................................................................................................................................................   21   0   LT-3
.......................uagaauuccauaacaauccauc............................................................................................................................................................................................   15   0   JCK-2
.......................uagaauucUauaacaauccauc............................................................................................................................................................................................   2    1   JCK-1
.......................uagaauuccauaacaauccGuca...........................................................................................................................................................................................   1    1   JCK-1
.......................uagaauuccauaacaauccauUa...........................................................................................................................................................................................   1    1   JCK-3
.......................uagaauuccauaacaauccauca...........................................................................................................................................................................................   8    0   LT-3
.......................uagaauuccauaacaauccaCca...........................................................................................................................................................................................   1    1   LT-1
.......................uagaauuccauaacaauccauUa...........................................................................................................................................................................................   3    1   LT-3
.......................uagaauuccauaacaauccauca...........................................................................................................................................................................................   29   0   JCK-3
.......................uagaauuccauaacaauccaucU...........................................................................................................................................................................................   1    1   JCK-3
.......................uagaauucUauaacaauccauca...........................................................................................................................................................................................   1    1   LT-1
.......................uagaauucAauaacaauccauca...........................................................................................................................................................................................   3    1   LT-3
.......................uCgaauuccauaacaauccauca...........................................................................................................................................................................................   1    1   JCK-3
.......................uagaauuccauaacaauccauca...........................................................................................................................................................................................   16   0   LT-1
.......................uagaauucUauaacaauccauca...........................................................................................................................................................................................   2    1   JCK-3
.......................uagaauuccauaacaauccaucU...........................................................................................................................................................................................   5    1   JCK-1
.......................uagaauuccauaacaGuccauca...........................................................................................................................................................................................   3    1   LCK-3
.......................uagaauuccauaacaauccauca...........................................................................................................................................................................................   13   0   LT-2
.......................uagaauuccauUacaauccauca...........................................................................................................................................................................................   1    1   LT-3
.......................uagCauuccauaacaauccauca...........................................................................................................................................................................................   1    1   JCK-1
.......................uagaauuccauaacaauccaucU...........................................................................................................................................................................................   2    1   JCK-2
.......................uagaauuccauaacaauccaucU...........................................................................................................................................................................................   1    1   LT-3
.......................uagaauuccauaacaauccauUa...........................................................................................................................................................................................   3    1   JCK-1
.......................uagaauuccauaacaauccauca...........................................................................................................................................................................................   21   0   JCK-2
.......................uagaauuccauaacaauccauca...........................................................................................................................................................................................   5    0   LCK-3
```

Mature Star

uuauaaauuccuaauggaguuuauagaauuccauaacaauccaucaaaucauuugaaguauauaauuuauuuuaaauuuauuaaagucuuagggggauguauucaauuuagaauuugauggauuuaaaauaaguuauagauuguaaaagauuugauagauuauuauagaauucuauugauuccauaaagauuuuauaagaauccaacgaaaucuuucguuuuaaggcugaauuuuauauuaacaauuuuc			
uagaauuccauaacaauccGuca	1	1	JCK-2
uagaauuccauaacaauccaucU	3	1	LT-1
uagaauucUauaacaauccauca	2	1	LT-3
uagaauucUauaacaauccauca	3	1	LCK-3
uagaauuccauaacaauccauca	38	0	JCK-1
uagaauucUauaacaauccauca	3	1	LT-2
uagaauucUauaacaauccauca	3	1	JCK-2
uagaauuccauaacaauccaucU	3	1	LT-2
uagaauuccauaacaaCccauca	1	1	JCK-1
uaAaauuccauaacaauccauca	3	1	JCK-1
uagaauuccauaacaauccauUa	1	1	JCK-2
uagaauuccauaacaaCccauca	2	1	JCK-3
uagaauuccauaacaauccaucaC	1	1	JCK-2
uagaauuccauaacaauccaucaa	1	0	JCK-1
uagaauuccauaacaauccaucaC	1	1	JCK-1
uagaauuccauaacaauccaucaa	3	0	JCK-2
uagaauucUauaacaauccaucaa	1	1	JCK-2
uagaCuuccauaacaauccaucaa	1	1	JCK-2
uagaauucUauaacaauccaucaa	1	1	JCK-3
uagaauuccauaacaauccaucaC	2	1	JCK-3
uagaauuccauaacaauccaucaa	2	0	JCK-3
uagaauuccauaacaauccaucaaU	1	1	JCK-3
uagaauuccauaacaauccaucaaU	1	1	JCK-1
agaauuccauaacaaucca	2	0	LT-2
agaauuccauaacaauccauU	1	1	LT-2
agaauuccauaacaauccauca	4	0	LT-2
agaauuccauaacaauccauca	2	0	LT-3
agaauuccauaacaauccaucaa	1	0	JCK-2
agaauuccauaacaauccaucaa	2	0	LT-3
agaauucUauaacaauccaucaa	3	1	LT-1
agaauuccauaacaauccaucaa	5	0	JCK-1
agaauucUauaacaauccaucaaa	1	1	LT-1
agaauuccauaacaauccaucaaa	1	0	LT-3
gaauuccauaacaauccaucaa	7	0	LT-2
aauuccauaacaauccauca	1	0	LT-1
aauuccauaacaauccaucaUa	1	1	JCK-3
aauuccauaacaauccaucaaauc	2	0	LT-1
aauucUauaacaauccaucaaauc	2	1	JCK-1
aauucUauaacaauccaucaaauc	1	1	LT-2
aauuccauUacaauccaucaaauc	1	1	JCK-1
aauuccauaGcaauccaucaaauc	1	1	LT-3
aauucUauaacaauccaucaaauc	2	1	JT-3
aauuccauUacaauccaucaaauc	1	1	JCK-3
aauuccauaacaauccaucaaauc	3	0	JCK-2
aauuccauaacaauccaucaaauc	3	0	JCK-1
aauuccauaacaauccaucaaauc	1	0	LT-2
aauucUauaacaauccaucaaauca	1	1	LT-2
auuccauaacaauccauc	1	0	LT-1
auuccauaacaauccaucaaauc	2	0	LT-2
uuccauaacaauccaucaaauc	1	0	LT-1
uuccauaacaGuccaucaaauc	1	1	LT-3
uuccauaacaauccaucaaaucau	1	0	JCK-2
uuccauaacaauccaucaaaucau	1	0	JCK-1
ucUauaacaauccaucaaauc	1	1	LT-3
uccauaacaauccaucaaauc	1	0	LT-3
ccauaacaauccaucaaauc	1	0	JCK-1
cauaacaauccaucaaauc	1	0	LT-1
cauaacaauccaucaaauc	1	0	LT-3
auaacaauccaucaaauc	5	0	LT-3
auaacaauccaucaaauc	11	0	LT-2
auaacaauccaucaaauc	2	0	LT-1
auaacaauccaucaaaucCuuug	3	1	JCK-2
auaacaauccaucaaaucCuuug	1	1	JCK-1
uaacaauccaucaaauUauu	3	1	LCK-3
uaacaauccaucaaaucCuuug	2	1	JCK-2
Uuuagggggauguauucaauuuag	1	1	JCK-3
Uuuagggggauguauucaauuuag	3	1	LT-1
Uuuagggggauguauucaauuuag	2	1	JCK-2
uuagggggauguauucaauuuaga	1	0	JCK-3
uagggggauguauucaauuuaga	8	0	LCK-3

Mature **Star**

uuauaaauuccuaauggaguuuauagaauuccauaacaauccaucaaaucauuugaaguauauaauuuauuuuaaauuuauuaaagucuuaggggauguauucaauuuagaauuugauggauuuaaaauaaguuauagauuguaaaagauuugauagauuauuauagaauucuauugauuccauaaagauuuuauaagaauccaacgaaaucuuucguuuuaaggcugaauuuuauauuaacaauuuuc

uagggggauguauucaauuuagaa	1	0	LT-3
aAggggauguauucaauuuaga	5	1	JT-2
aAggggauguauucaauuuaga	10	1	JT-3
agAgggauguauucaauuuagaa	7	1	LCK-3
agggggauguauucaauuuagaCu	1	1	JCK-2
agggggauguauucaauuuagaau	1	0	LT-1
agggggauguauucaauuuagaGu	1	1	LT-1
agAgggauguauucaauuuagaau	3	1	JCK-1
agggAgauguauucaauuuagaau	1	1	JCK-3
agggggauguauucaauuuagaau	1	0	JCK-1
agggAgauguauucaauuuagaau	2	1	JCK-2
agggggauguauucaauuuagaau	1	0	LT-2
agggggauguauucaauuuagaCu	2	1	JCK-3
aAggggauguauucaauuuagaau	2	1	LT-1
agAgggauguauucaauuuagaau	3	1	JCK-2
gAgggauguauucaauuuagaau	1	1	LT-2
gggggauguauucaauuuagaau	2	0	LT-2
gggggauguauucaauuuagaau	2	0	LCK-3
gggggCuguauucaauuuagaauu	1	1	JCK-1
gggggauguauuUaauuuagaauu	1	1	JCK-1
ggggauguauucaauuuagaG	1	1	LT-3
ggggauguauuUaauuuagaau	1	1	LT-2
ggggauguauucaauuuagaau	3	0	LT-2
Agggauguauucaauuuagaau	2	1	LT-1
ggggauguauucaauuuagaau	1	0	LT-3
ggggauguauucaauuuagaau	1	0	JCK-2
ggggauguauucaauuuagaGu	3	1	LCK-3
ggggauguauucaauuuagaGuu	1	1	JCK-2
ggggauguauucaauuuagaauuu	2	0	JCK-3
gggauguauucaauuuaga	1	0	LT-3
gggauguauucaauuuagaauuug	2	0	LT-3
gggauguauucaauuuagaauuug	3	0	JCK-2
gggauguauucaauuuagaauuug	1	0	JCK-1
Cgauguauucaauuuagaau	4	1	LCK-3
guauucGauuuagaauuugaugga	1	1	JCK-3
ucaauuuagaauuugauggau	2	0	LT-1
caauuuagaauuugaugga	2	0	LT-1
caauuuagaauuuAaugga	5	1	LCK-3
caauuuagaauuuAaugga	2	1	LT-1
caauuuagaauuugaugga	6	0	LT-2
caauuuagaauuugauggauu	1	0	LT-2
caauuuagaauuugauggauu	1	0	LT-3
caauuuagaauuugauggauuu	1	0	LT-3
caauuuagaauuuAauggauuu	1	1	LT-2
aauuuagaauuugaugga	1	0	LT-1
aauuuagaauuugauggauu	1	0	LT-1
aauuuagaauuugauggauuuaa	1	0	JT-3
aauuuagaauuugauggauuuaa	2	0	JT-2
auuuagaauuugauggauu	5	0	LCK-3
auuuagaCuuugauggauuua	1	1	JCK-2
auuuagaCuuugauggauuua	3	1	JCK-3
auuuagaCuuugauggauuua	1	1	JCK-1
agaauuugauggauuuaaaauGag	3	1	LT-3
agaauuugauggauuuaaaauGag	2	1	JCK-3
agaauuugauggauuuaaaauGag	2	1	LT-1
agaauuugauggauuuaaaauGag	2	1	JCK-1
agaauuugauggauuuaaaauGag	6	1	JCK-2
gaauuugauggauuuaaaauGag	1	1	LT-3
auuugauggauuuaaaauGaguua	2	1	JCK-1
uugauggauuuaaaaGaagu	2	1	JCK-2
uaaaagauuugauGgauuau	1	1	LT-1
aaaGgauuugauagauuauu	2	1	JCK-2
aaaGgauuugauagauuauu	1	1	JCK-3
aaaGgauuugauagauuauuau	1	1	JCK-2
aaaGgauuugauagauuauuau	2	1	JCK-3
aaaagauuugauagauuauuauGg	3	1	LT-2
aaagauuugauagauuauuau	1	0	JCK-2
aaagauuugauagauuauuau	2	0	JCK-3
aaagauuugauagauuauuau	1	0	JCK-1
aaagauuugauagauuGuuaua	1	1	LT-1

```
                    Mature                                                                                                                    Star
uuauaaauuccuaauggaguuuauagaauuccauaacaauccaucaaaucauuugaaguauauaauuuauuuuaaauuuauuaaagucuuagggggauguauucaauuuagaauuugauggauuuaaaauaaguuauagauuguaaaagauuugauagauuauuauagaauucuauugauuccauaaagauuuuauaagaauccaacgaaaucuuucguuuuaaggcugaauuuuauauuaacaauuuuc
..................................................................................................................................................aGgauuugauagauuauuauagaa................................................................................   1   1   LT-3
.....................................................................................................................................................auuugauagauuGuuauagaauuc.............................................................................   1   1   JCK-3
.......................................................................................................................................................................................auaaagauuGuauaagaau................................................   1   1   LT-1
..................................................................................................................................................................................................aAaagaauccaacgaaaucu....................................   1   1   LT-1
...........................................................................................................................................................................................................................uuuaagAcugaauuuuauauu..........   1   1   LT-2
............................................................................................................................................................................................................................uuaaggcugGauuuuauauu..........   1   1   LT-3
.............................................................................................................................................................................................................................uaaggcugaauuAuauauuaac.......   1   1   LT-1
```
